# *Notes from the Field:* Outbreak of COVID-19 Among a Highly Vaccinated Population Aboard a U.S. Navy Ship After a Port Visit — Reykjavik, Iceland, July 2021

**DOI:** 10.15585/mmwr.mm7107a5

**Published:** 2022-02-18

**Authors:** Tammy E. Servies, Eric C. Larsen, Rodney C. Lindsay, Jonathan S. Jones, Regina Z. Cer, Logan J. Voegtly, Matthew R. Lueder, Francisco Malagon, Kimberly A. Bishop-Lilly, Asha J. Riegodedios

**Affiliations:** ^1^Navy Environmental and Preventive Medicine Unit 7, Rota, Spain; ^2^Naval Medical Research Center – Frederick, Fort Detrick, Maryland; ^3^Leidos, Reston, Virginia; ^4^Navy and Marine Corps Public Health Center, Portsmouth, Virginia.

On July 27, 2021, a fully vaccinated* crew member on a U.S. Navy ship who had been symptomatic with cough and congestion for 4 days was evaluated in the ship’s onboard medical department and received a positive test result^†^ for SARS-CoV-2, the virus that causes COVID-19. The ship had approximately 350 personnel on board^§^; COVID-19 vaccination rate was >98%.^¶^ The ship had been on an 8-week deployment with port visits in Norway (July 13–14) and in Reykjavik, Iceland (July 18–21). Masking and physical distancing mandates on the ship were relaxed while at sea but were immediately reimplemented upon identification of the crew member’s positive test result. During the deployment, personnel had permission to go ashore only during the Iceland port visit and only if they were fully vaccinated. Before July 27, no one had been evaluated at the onboard medical department for respiratory symptoms. Although reported COVID-19 incidence was low in Iceland just before the port visit (17.5 per 100,000 population on July 18), incidence increased approximately elevenfold, to 219.5 per 100,000 on July 27 with emergence of the B.1.617.2 (Delta) variant.** At the onset of the COVID-19 pandemic, outbreaks on some U.S. Navy ships led to attack rates greater than 25% (*1*) of the crew in the confined environment. In this outbreak during Delta variant predominance, the combination of a high vaccination rate with prevention strategies resulted in a lower (6.3%) attack rate of COVID-19 than seen at the onset of the pandemic.

After identification of the initial case on July 27, all ship personnel were notified to report to the onboard medical department if they had any COVID-19–like signs or symptoms,^††^ resulting in diagnoses of an additional 11 COVID-19 cases that day. The ship immediately instituted prevention measures, including mask use, physical distancing, increased cleaning, isolation of the 12 initial patients, testing of 69 close contacts,^§§^ and testing and quarantine of six unvaccinated persons (two of whom were also close contacts). On July 28 and 29, six additional cases were identified through testing. Nasal swabs from these 18 persons with positive antigen test results were sent off the ship for reverse transcription–polymerase chain reaction (RT-PCR) testing and all were positive for SARS-CoV-2.^¶¶^ Further analysis determined 17 of the 18 specimens were Delta variant AY.9 lineage; 16 of the 17 were identical.*** During this same time frame at the end of July, Delta AY.9 was identified in 8% of specimens in Iceland and fewer than 1% of specimens in Norway and the United States.^†††^ The 18 infected persons were removed from the ship on July 31 to reduce the ship’s health care requirements and to prevent further transmission. Four additional cases of COVID-19 were identified during August 1–7 (including three diagnosed aboard the ship and one postdeployment) with onset July 28–August 5. The overall attack rate was 6.3%. The ship returned to its home port on August 3, concluding its deployment as scheduled.

Among the 22 infected personnel identified, all were fully vaccinated, and all were symptomatic. Most (91%) were aged <40 years (average age = 30.2 years). No patient required hospitalization or supplemental oxygen and no deaths occurred. Before the outbreak was identified on July 27, 13 (59%) of the 22 infected personnel had been symptomatic for a median of 3 days (range = 1–5 days) aboard the ship with no masking or physical distancing protocols in place (Figure). During the 15-day outbreak period (July 22–August 5), 91 personnel received rapid antigen testing.

**FIGURE:**
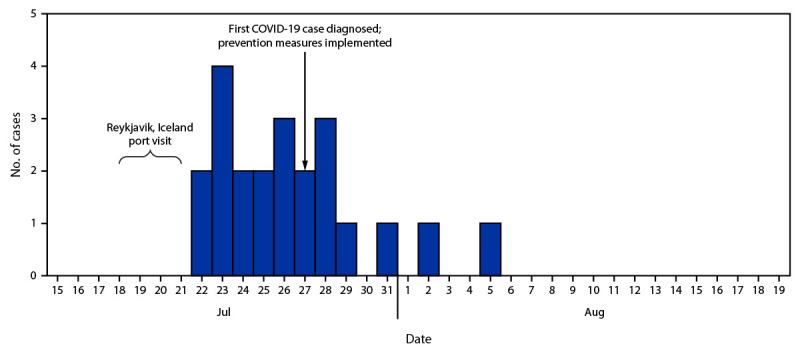
Date of symptom onset or specimen collection* for COVID-19 cases identified during an outbreak on a U.S. Navy ship (N = 22) — Reykjavik, Iceland, July–August 2021^†^ * Whichever occurred earlier; for all but one case, symptom onset preceded specimen collection. ^†^ Prevention measures included mask use, physical distancing, increased cleaning, canvassing for mild symptoms, and increased testing.

Only one case was identified >14 days after the Iceland port visit, demonstrating very limited spread of infection despite exposure to symptomatic personnel for a median of 3 days in the confined shipboard spaces. In previous U.S. Navy shipboard outbreaks, before COVID-19 vaccines were available, SARS-CoV-2 spread was rapid and extensive, with attack rates of 26.6% (1,271 of 4,779 personnel) on one ship (*1*) and 36.3% (121 of 333) on another (Navy and Marine Corps Public Health Center, unpublished data, 2020). These attack rates were approximately four and six times higher, respectively, than that described in this report.

The findings in this report are subject to at least four limitations. First, shipboard testing was limited to rapid antigen testing, which has a lower sensitivity than RT-PCR testing in asymptomatic persons (*2*). Second, testing relied on persons to report symptoms and close contacts, which is subject to recall bias. Third, this was an outbreak of Delta variant and findings might not be applicable to B.1.1.529 (Omicron) or other variant outbreaks. Finally, this outbreak occurred in a highly vaccinated, young, healthy population, thus limiting generalizability to the overall U.S. population.

This outbreak in the enclosed environment of a ship suggests that high vaccination rates, in combination with COVID-19 prevention measures, can substantially reduce the spread of SARS-CoV-2, despite the high transmissibility of the Delta variant and introduction of SARS-CoV-2 into a congregate setting. Infections among vaccinated persons did occur, which is expected (*3*), but symptoms were mild. Vaccination, in coordination with multicomponent prevention strategies, are critical to limiting SARS-CoV-2 transmission and COVID-19–related illness.

## Summary

### What is already known about this topic?

At the onset of the COVID-19 pandemic, outbreaks on some U.S. Navy ships led to attack rates >25% in the confined environment.

### What is added by this report?

During July 2021, an outbreak of Delta variant aboard a U.S. Navy ship after a port visit in Iceland resulted in a 6% attack rate. The ship’s population was >98% immunized, and although prevention measures (e.g., mask use, extra cleaning, and distancing procedures) were relaxed during the underway period, they were reimplemented upon identification of the first case.

### What are the implications for public health practice?

Vaccination, in combination with other prevention strategies, resulted in a much lower attack rate of COVID-19 than seen in the early months of the pandemic.

## Footnotes

* Fully vaccinated was defined as 2 weeks after receipt of a single dose of Ad.26.COV2.S (Janssen [Johnson & Johnson]) vaccine or the second dose of either BNT162b2 (Pfizer-BioNTech) or mRNA-1273 (Moderna) vaccines.

† The Abbott BinaxNOW COVID-19 Ag Card rapid antigen test was used to test personnel aboard the ship. During the deployment, personnel evaluated at the clinic for COVID-19–like symptoms were tested when seen by the ship’s medical department.

§ During July 18–August 3, the total number of crew members fluctuated between 346 and 355.

¶ This outbreak occurred 1 month before the August 24, 2021, memo by the U.S. Secretary of Defense mandating vaccines in service members. However, personnel were not authorized to depart the ship for liberty without being fully vaccinated.

** https://www.covid.is/data/ (Accessed December 16, 2021)

†† Fever, chills, rigors, myalgia, headache, sore throat, loss of taste or smell, cough, shortness of breath, or difficulty breathing.

§§ A close contact was defined as anyone within 6 feet of an infected person for a cumulative total of ≥15 minutes within a 24-hour period.

¶¶ Samples were sent to the U.S. Naval Hospital in Rota, Spain, for RT-PCR testing and then sent to Naval Medical Research Center – Frederick on Fort Detrick, Maryland, for genome sequencing and phylogenetic analysis.

*** One of the 17 Delta variant samples had an additional mutation in ORF10 (G29645T, ORF10) resulting in a lysine versus a valine at amino acid V30L. The final sample was not assigned a lineage because of insufficient consensus genome length.

††† Reported data from Outbreak.info’s AY.9 Lineage Report. https://outbreak.info/situation-reports?pango%C2%A0=%C2%A0AY.9&loc%C2%A0=%C2%A0ISL&loc%C2%A0=%C2%A0NOR&loc%C2%A0=%C2%A0USA&selected%C2%A0=%C2%A0ISL&overlay%C2%A0=%C2%A0false (Accessed December 31, 2021).
